# Women and Their Duty

**Published:** 1892-03

**Authors:** 


### WOMEN AND THEIR DUTY.

Dr. Arabella Kenealy, a London physician of wide experience, has this to say in regard to women and professions: Women should not attempt to carry on a profession after marriage. I mean the women of the upper and middle classes who go into the professions. It is not necessary that they should be the bread-winners; that duty should devolve upon the husband, and I am confident that the rising generation would be healthier and stronger in every way if the mothers would exert themselves less. I look anxiously at every baby that comes under my notice in the hope that I shall find some improvement in the type, some increase in stamina, compared with the generation that has preceded it; but instead of this there is only deterioration observable. This deterioration is particularly noticeable among the children of very active mothers. The cleverest and most highly educated women, the women who take the most active part in public affairs, have the most weakly and puny children. Another thing, women are going into too active forms of exercise. When a young married woman tells me that she is captain of a cricket eleven or a football team I can only say I am perfectly aghast. Women must place before themselves the alternative, to earn their living, to exercise their faculties, and to gratify their ambitions in a professional career, or to become good wives and mothers, and if they choose the domestic life they must recognize that they must sacrifice their personal happiness and ambition in the happiness and success of their children.

---

To Hall's Journal of Health:

Sir:—You have for some months advertised our Herba Vita. We are glad to be able to say that we have received many orders for it which are due to your agency, not a few from practicing physicians several times repeated.

In the course of an extensive business of some years, extending over this and foreign lands, we have yet to hear the first complaint that Herba Vita does not accomplish all that is claimed for it, and Hall's Journal and all other publications advertising this Remedy are authorized to state that any person purchasing it for any of the disorders for which it is recommended, and finding it ineffectual, is authorized to return the package and the amount paid for it will be refunded. As a preventive or cure of "La Grippe," this remedy is incomparable.

Herba Vita Remedy Co.,
340 W. 59th street.

Office, February 23d, 1892.

We have used and recommended Herba Vita to many of our friends, and feel confident that the proprietors will seldom, if ever, be called upon to refund its cost. A small package costing 25 cents will often save as many dollars, and keep the doctor from the door. [Ed.

